# Supplementary material for: Development of a computer-aided design software for the quantitative evaluation of aesthetic damage
Source: PLoS One. 2019 Dec 18;14(12):e0226322. doi: 10.1371/journal.pone.0226322 (PMC6919621; doi:10.1371/journal.pone.0226322)
Supplement: S1 Code site — 1. (ZIP) [file pone.0226322.s001.zip › [EESC_jr][Projeto]Relatorio_07_21/reports/templates_zip/gentelella-master.zip!/gentelella-master/vendors/DateJS/reports/lcov-report/core/sugarpak.js.html]

Code coverage report for core/sugarpak.js


# Code coverage report for core/sugarpak.js

## Statements: 96.62% (143 / 148)      Branches: 82.86% (58 / 70)      Functions: 100% (30 / 30)      Lines: 96.58% (141 / 146)      Ignored: none

All files » core/ » sugarpak.js

```
|  |  |  |
| --- | --- | --- |
| 1 2 3 4 5 6 7 8 9 10 11 12 13 14 15 16 17 18 19 20 21 22 23 24 25 26 27 28 29 30 31 32 33 34 35 36 37 38 39 40 41 42 43 44 45 46 47 48 49 50 51 52 53 54 55 56 57 58 59 60 61 62 63 64 65 66 67 68 69 70 71 72 73 74 75 76 77 78 79 80 81 82 83 84 85 86 87 88 89 90 91 92 93 94 95 96 97 98 99 100 101 102 103 104 105 106 107 108 109 110 111 112 113 114 115 116 117 118 119 120 121 122 123 124 125 126 127 128 129 130 131 132 133 134 135 136 137 138 139 140 141 142 143 144 145 146 147 148 149 150 151 152 153 154 155 156 157 158 159 160 161 162 163 164 165 166 167 168 169 170 171 172 173 174 175 176 177 178 179 180 181 182 183 184 185 186 187 188 189 190 191 192 193 194 195 196 197 198 199 200 201 202 203 204 205 206 207 208 209 210 211 212 213 214 215 216 217 218 219 220 221 222 223 224 225 226 227 228 229 230 231 232 233 234 235 236 237 238 239 240 241 242 243 244 245 246 247 248 249 250 251 252 253 254 255 256 257 258 259 260 261 262 263 264 265 266 267 268 269 270 271 272 273 274 275 276 277 278 279 280 281 282 283 284 285 286 287 288 289 290 291 292 293 294 295 296 297 298 299 300 301 302 303 304 305 306 307 308 309 310 311 312 313 314 315 316 317 318 319 320 321 322 323 324 325 326 327 328 329 330 331 332 333 334 335 336 337 338 339 340 341 342 343 344 345 346 347 348 349 350 351 352 353 354 355 356 357 358 359 360 361 362 363 364 365 366 367 368 369 370 371 372 373 374 375 376 377 378 379 380 381 382 383 384 385 386 387 388 389 390 391 392 393 394 395 396 397 398 399 400 401 402 403 404 405 406 407 408 409 410 411 412 413 414 415 416 417 418 419 420 421 422 423 424 425 426 427 428 429 430 431 432 433 434 435 436 437 438 439 440 441 442 443 444 445 446 447 448 449 450 451 452 453 454 455 456 457 458 459 460 461 462 463 464 465 466 467 468 469 470 471 472 473 474 475 476 477 478 479 480 481 482 483 484 485 486 487 488 489 490 491 492 493 494 | 1 1     1     1     1     1     1     1                             1 24 24 24                               1 7                               1 1137 1137 1137                               1 7                             1 34 34                                                                           1 5 5 5                             1 3                       1 13 1   12 4   8 7 7   1                     1 3 2 2   1                               1 1                                   1 1 1 1                                   1 1   1 1         1                                             1 10 10 100 90     10                                 1 1 1         1 8 1179 20 20   1159 1159                         4 3     4   4 4 4 4 4 1   3   1155       1 7 8 8     8             1 12 17 2 2   15       1 12 12       1 2   19   19   19         1 1     1 10     89         89 5 5           5   5 31 31 1   30 4     4     84 7   84 84         1 9 2 2       1 10 10   9     9       1   1 6 5     5     5     5 4 4   1       1 6 | ``` /*************************************************************  * SugarPak - Domain Specific Language -  Syntactical Sugar  *  *************************************************************/   (function () { 	var $D = Date, $P = $D.prototype, $N = Number.prototype;   	// private 	$P._orient = +1;   	// private 	$P._nth = null;   	// private 	$P._is = false;   	// private 	$P._same = false; 	 	// private 	$P._isSecond = false;   	// private 	$N._dateElement = "days";   	/**  	 * Moves the date to the next instance of a date as specified by the subsequent date element function (eg. .day(), .month()), month name function (eg. .january(), .jan()) or day name function (eg. .friday(), fri()). 	 * Example 	<pre><code> 	Date.today().next().friday(); 	Date.today().next().fri(); 	Date.today().next().march(); 	Date.today().next().mar(); 	Date.today().next().week(); 	</code></pre> 	 *  	 * @return {Date}    date 	 */ 	$P.next = function () { 		this._move = true; 		this._orient = +1; 		return this; 	};   	/**  	 * Creates a new Date (Date.today()) and moves the date to the next instance of the date as specified by the subsequent date element function (eg. .day(), .month()), month name function (eg. .january(), .jan()) or day name function (eg. .friday(), fri()). 	 * Example 	<pre><code> 	Date.next().friday(); 	Date.next().fri(); 	Date.next().march(); 	Date.next().mar(); 	Date.next().week(); 	</code></pre> 	 *  	 * @return {Date}    date 	 */ 	$D.next = function () { 		return $D.today().next(); 	};   	/**  	 * Moves the date to the previous instance of a date as specified by the subsequent date element function (eg. .day(), .month()), month name function (eg. .january(), .jan()) or day name function (eg. .friday(), fri()). 	 * Example 	<pre><code> 	Date.today().last().friday(); 	Date.today().last().fri(); 	Date.today().last().march(); 	Date.today().last().mar(); 	Date.today().last().week(); 	</code></pre> 	 *   	 * @return {Date}    date 	 */ 	$P.last = $P.prev = $P.previous = function () { 		this._move = true; 		this._orient = -1; 		return this; 	};   	/**  	 * Creates a new Date (Date.today()) and moves the date to the previous instance of the date as specified by the subsequent date element function (eg. .day(), .month()), month name function (eg. .january(), .jan()) or day name function (eg. .friday(), fri()). 	 * Example 	<pre><code> 	Date.last().friday(); 	Date.last().fri(); 	Date.previous().march(); 	Date.prev().mar(); 	Date.last().week(); 	</code></pre> 	 *   	 * @return {Date}    date 	 */ 	$D.last = $D.prev = $D.previous = function () { 		return $D.today().last(); 	};   	/**  	 * Performs a equality check when followed by either a month name, day name or .weekday() function. 	 * Example 	<pre><code> 	Date.today().is().friday(); // true\|false 	Date.today().is().fri(); 	Date.today().is().march(); 	Date.today().is().mar(); 	</code></pre> 	 *   	 * @return {Boolean}    true\|false 	 */ 	$P.is = function () { 		this._is = true; 		return this; 	};   	/**  	 * Determines if two date objects occur on/in exactly the same instance of the subsequent date part function. 	 * The function .same() must be followed by a date part function (example: .day(), .month(), .year(), etc). 	 * 	 * An optional Date can be passed in the date part function. If now date is passed as a parameter, 'Now' is used.  	 * 	 * The following example demonstrates how to determine if two dates fall on the exact same day. 	 * 	 * Example 	<pre><code> 	var d1 = Date.today(); // today at 00:00 	var d2 = new Date();   // exactly now.   	// Do they occur on the same day? 	d1.same().day(d2); // true 	 	// Do they occur on the same hour? 	d1.same().hour(d2); // false, unless d2 hour is '00' (midnight). 	 	// What if it's the same day, but one year apart? 	var nextYear = Date.today().add(1).year();   	d1.same().day(nextYear); // false, because the dates must occur on the exact same day.  	</code></pre> 	 * 	 * Scenario: Determine if a given date occurs during some week period 2 months from now.  	 * 	 * Example 	<pre><code> 	var future = Date.today().add(2).months(); 	return someDate.same().week(future); // true\|false; 	</code></pre> 	 *   	 * @return {Boolean}    true\|false 	 */ 	$P.same = function () { 		this._same = true; 		this._isSecond = false; 		return this; 	};   	/**  	 * Determines if the current date/time occurs during Today. Must be preceded by the .is() function. 	 * Example 	<pre><code> 	someDate.is().today();    // true\|false 	new Date().is().today();  // true 	Date.today().is().today();// true 	Date.today().add(-1).day().is().today(); // false 	</code></pre> 	 *   	 * @return {Boolean}    true\|false 	 */ 	$P.today = function () { 		return this.same().day(); 	};   	/**  	 * Determines if the current date is a weekday. This function must be preceded by the .is() function. 	 * Example 	<pre><code> 	Date.today().is().weekday(); // true\|false 	</code></pre> 	 *   	 * @return {Boolean}    true\|false 	 */ 	$P.weekday = function () { 		if (this._nth) { 			return df("Weekday").call(this); 		} 		if (this._move) { 			return this.addWeekdays(this._orient); 		} 		if (this._is) { 			this._is = false; 			return (!this.is().sat() && !this.is().sun()); 		} 		return false; 	}; 	/**  	 * Determines if the current date is on the weekend. This function must be preceded by the .is() function. 	 * Example 	<pre><code> 	Date.today().is().weekend(); // true\|false 	</code></pre> 	 *   	 * @return {Boolean}    true\|false 	 */ 	$P.weekend = function () { 		if (this._is) { 			this._is = false; 			return (this.is().sat() \|\| this.is().sun()); 		} 		return false; 	};   	/**  	 * Sets the Time of the current Date instance. A string "6:15 pm" or config object {hour:18, minute:15} are accepted. 	 * Example 	<pre><code> 	// Set time to 6:15pm with a String 	Date.today().at("6:15pm");   	// Set time to 6:15pm with a config object 	Date.today().at({hour:18, minute:15}); 	</code></pre> 	 *   	 * @return {Date}    date 	 */ 	$P.at = function (time) { 		return (typeof time === "string") ? $D.parse(this.toString("d") + " " + time) : this.set(time); 	}; 		 	/**  	 * Creates a new Date() and adds this (Number) to the date based on the preceding date element function (eg. second\|minute\|hour\|day\|month\|year). 	 * Example 	<pre><code> 	// Undeclared Numbers must be wrapped with parentheses. Requirment of JavaScript. 	(3).days().fromNow(); 	(6).months().fromNow();   	// Declared Number variables do not require parentheses.  	var n = 6; 	n.months().fromNow(); 	</code></pre> 	 *   	 * @return {Date}    A new Date instance 	 */ 	$N.fromNow = $N.after = function (date) { 		var c = {}; 		c[this._dateElement] = this; 		return ((!date) ? new Date() : date.clone()).add(c); 	};   	/**  	 * Creates a new Date() and subtract this (Number) from the date based on the preceding date element function (eg. second\|minute\|hour\|day\|month\|year). 	 * Example 	<pre><code> 	// Undeclared Numbers must be wrapped with parentheses. Requirment of JavaScript. 	(3).days().ago(); 	(6).months().ago();   	// Declared Number variables do not require parentheses.  	var n = 6; 	n.months().ago(); 	</code></pre> 	 *   	 * @return {Date}    A new Date instance 	 */ 	$N.ago = $N.before = function (date) { 		var c = {}, 		s = (this._dateElement[this._dateElement.length-1] !== "s") ? this._dateElement + "s" : this._dateElement; 		c[s] = this * -1; 		return ((!date) ? new Date() : date.clone()).add(c); 	};   	// Do NOT modify the following string tokens. These tokens are used to build dynamic functions. 	// All culture-specific strings can be found in the CultureInfo files. 	var dx = ("sunday monday tuesday wednesday thursday friday saturday").split(/\s/), 		mx = ("january february march april may june july august september october november december").split(/\s/), 		px = ("Millisecond Second Minute Hour Day Week Month Year Quarter Weekday").split(/\s/), 		pxf = ("Milliseconds Seconds Minutes Hours Date Week Month FullYear Quarter").split(/\s/), 		nth = ("final first second third fourth fifth").split(/\s/), 		de;      /**  	 * Returns an object literal of all the date parts. 	 * Example 	<pre><code> 	var o = new Date().toObject(); 	 	// { year: 2008, month: 4, week: 20, day: 13, hour: 18, minute: 9, second: 32, millisecond: 812 } 	 	// The object properties can be referenced directly from the object. 	 	alert(o.day);  // alerts "13" 	alert(o.year); // alerts "2008" 	</code></pre> 	 *   	 * @return {Date}    An object literal representing the original date object. 	 */ 	$P.toObject = function () { 		var o = {}; 		for (var i = 0; i < px.length; i++) { 			if (this["get" + pxf[i]]) { 				o[px[i].toLowerCase()] = this["get" + pxf[i]](); 			} 		} 		return o; 	};        /**  	 * Returns a date created from an object literal. Ignores the .week property if set in the config.  	 * Example 	<pre><code> 	var o = new Date().toObject(); 	 	return Date.fromObject(o); // will return the same date.    	var o2 = {month: 1, day: 20, hour: 18}; // birthday party! 	Date.fromObject(o2); 	</code></pre> 	 *   	 * @return {Date}    An object literal representing the original date object. 	 */ 	$D.fromObject = function(config) { 		config.week = null; 		return Date.today().set(config); 	}; 		 	// Create day name functions and abbreviated day name functions (eg. monday(), friday(), fri()). 	 	var df = function (n) { 		return function () { 			if (this._is) { 				this._is = false; 				return this.getDay() === n; 			} 			if (this._move) { this._move = null; } 			if (this._nth !== null) { 				// If the .second() function was called earlier, remove the _orient  				// from the date, and then continue. 				// This is required because 'second' can be used in two different context. 				//  				// Example 				// 				//   Date.today().add(1).second(); 				//   Date.march().second().monday(); 				//  				// Things get crazy with the following... 				//   Date.march().add(1).second().second().monday(); // but it works!! 				//   				if (this._isSecond) { 					this.addSeconds(this._orient * -1); 				} 				// make sure we reset _isSecond 				this._isSecond = false;   				var ntemp = this._nth; 				this._nth = null; 				var temp = this.clone().moveToLastDayOfMonth(); 				this.moveToNthOccurrence(n, ntemp); 				if (this > temp) { 					throw new RangeError($D.getDayName(n) + " does not occur " + ntemp + " times in the month of " + $D.getMonthName(temp.getMonth()) + " " + temp.getFullYear() + "."); 				} 				return this; 			} 			return this.moveToDayOfWeek(n, this._orient); 		}; 	}; 	 	var sdf = function (n) { 		return function () { 			var t = $D.today(), shift = n - t.getDay(); 			Iif (n === 0 && Date.CultureInfo.firstDayOfWeek === 1 && t.getDay() !== 0) { 				shift = shift + 7; 			} 			return t.addDays(shift); 		}; 	}; 	   	 	// Create month name functions and abbreviated month name functions (eg. january(), march(), mar()). 	var month_instance_functions = function (n) { 		return function () { 			if (this._is) { 				this._is = false; 				return this.getMonth() === n; 			} 			return this.moveToMonth(n, this._orient); 		}; 	}; 	 	var month_static_functions = function (n) { 		return function () { 			return $D.today().set({ month: n, day: 1 }); 		}; 	}; 	 	var processTerms = function (names, staticFunc, instanceFunc) { 		for (var i = 0; i < names.length; i++) { 			// Create constant static Name variables. 			$D[names[i].toUpperCase()] = $D[names[i].toUpperCase().substring(0, 3)] = i; 			// Create Name functions. 			$D[names[i]] = $D[names[i].substring(0, 3)] = staticFunc(i); 			// Create Name instance functions. 			$P[names[i]] = $P[names[i].substring(0, 3)] = instanceFunc(i); 		}   	};   	processTerms(dx, sdf, df); 	processTerms(mx, month_static_functions, month_instance_functions); 	 	// Create date element functions and plural date element functions used with Date (eg. day(), days(), months()). 	var ef = function (j) { 		return function () { 			// if the .second() function was called earlier, the _orient  			// has alread been added. Just return this and reset _isSecond. 			Iif (this._isSecond) { 				this._isSecond = false; 				return this; 			}   			if (this._same) { 				this._same = this._is = false; 				var o1 = this.toObject(), 					o2 = (arguments[0] \|\| new Date()).toObject(), 					v = "", 					k = j.toLowerCase();   				// the substr trick with -1 doesn't work in IE8 or less 				k = (k[k.length-1] === "s") ? k.substring(0,k.length-1) : k; 					 				for (var m = (px.length - 1); m > -1; m--) { 					v = px[m].toLowerCase(); 					if (o1[v] !== o2[v]) { 						return false; 					} 					if (k === v) { 						break; 					} 				} 				return true; 			} 			 			if (j.substring(j.length - 1) !== "s") { 				j += "s"; 			} 			if (this._move) { this._move = null; } 			return this["add" + j](this._orient); 		}; 	}; 	 	 	var nf = function (n) { 		return function () { 			this._dateElement = n; 			return this; 		}; 	};     	for (var k = 0; k < px.length; k++) { 		de = px[k].toLowerCase(); 		if(de !== "weekday") { 			// Create date element functions and plural date element functions used with Date (eg. day(), days(), months()). 			$P[de] = $P[de + "s"] = ef(px[k]); 			 			// Create date element functions and plural date element functions used with Number (eg. day(), days(), months()). 			$N[de] = $N[de + "s"] = nf(de + "s"); 		} 	} 	 	$P._ss = ef("Second"); 	 	var nthfn = function (n) { 		return function (dayOfWeek) { 			Iif (this._same) { 				return this._ss(arguments[0]); 			} 			Iif (dayOfWeek \|\| dayOfWeek === 0) { 				return this.moveToNthOccurrence(dayOfWeek, n); 			} 			this._nth = n;   			// if the operator is 'second' add the _orient, then deal with it later... 			if (n === 2 && (dayOfWeek === undefined \|\| dayOfWeek === null)) { 				this._isSecond = true; 				return this.addSeconds(this._orient); 			} 			return this; 		}; 	};   	for (var l = 0; l < nth.length; l++) { 		$P[nth[l]] = (l === 0) ? nthfn(-1) : nthfn(l); 	} }());   ``` |
```

Generated by istanbul at Sun Nov 16 2014 12:08:33 GMT-0800 (PST)
